# A Novel System to Monitor Tic Attacks for Tourette Syndrome Using Machine Learning and Wearable Technology: Preliminary Survey Study and Proposal for a New Sensing Device

**DOI:** 10.2196/43351

**Published:** 2023-04-25

**Authors:** Agni Rajinikanth, Davis Kevin Clark, Marianna Evangelia Kapsetaki

**Affiliations:** 1 Rutgers Preparatory School Somerset, NJ United States; 2 St George's School Vancouver, BC Canada; 3 Faculty of Life Sciences Division of Biosciences University College London London United Kingdom

**Keywords:** Tourette syndrome, neurological diseases, tic attacks, wearable technology, movement disorders, tremor monitoring, biosensing technology, automatic tic detection

## Abstract

**Background:**

Tourette syndrome is a neurological disorder that is characterized by repeated unintentional physical movement and vocal sounds, better known as tics. Cases of mild Tourette can have tics numerous times throughout the day, while severe cases may have tics every 5 to 10 seconds. At certain times, typically during high levels of stress, tics become chained in an incessant, continuous fashion—this is known as a tic attack. Tic attacks incapacitate the patient, rendering it difficult for them to move, perform daily actions, and even communicate with others. Caretakers—usually guardians, family members, or nurses—can help reduce the time tic attacks last with their presence and by providing emotional support to the patient.

**Objective:**

We describe TSBand, a wearable wristband that uses machine learning algorithms and a variety of sensors to monitor for tic attacks and notify caretakers when an attack occurs.

**Methods:**

We conducted a research survey with 70 Tourette patients to determine the usability and functionality of TSBand; internal review board approval was not required.

**Results:**

This study has resulted in a smart wristband prototype that costs US $62.74; it uses movement, heart rate, sweat, and body temperature to detect tic attacks using a hybrid local outlier factoring and regression algorithm. An audio tic attack detection mechanism is also included, using recurrent neural networks, and a manually activated backup button and backup audio mechanism are fitted to alert caretakers on the personalized companion app.

**Conclusions:**

TSBand enables the caretaker to provide support faster and prevent excessive self-harm or injury during the attack. It is an affordable and effective solution, solving a problem that many Tourette patients, often children, face. This study has not had the opportunity to test TSBand with any Tourette patients, and we aim to perform rigorous testing and analysis after grant funding is secured.

## Introduction

### Background

An estimated 350,000 to 450,000 children and adults in the United States alone have Tourette syndrome (TS), a neurological disorder that causes involuntary movements or vocal sounds known as tics, and about 1 million children and adults in the United States have other persistent tic disorders [1]. Tics are usually most prevalent in adolescents, and the severity of the disorder tends to decrease with age, making children with TS a community that needs medical and technological innovation. Research has shown that around 1% to 3% of children in mainstream schools are affected by TS [2]. Specific tics and their severity vary from person to person (eg, hand gestures vs whole-arm movements), but nearly all tics can be categorized as uncontrollable movements or audible noises. Specific tics can both develop and disappear at random. People who have TS often also experience symptoms of obsessive-compulsive disorder and attention deficit hyperactivity disorder in addition to tics [3].

Under certain situations of high stress or anxiety, a tic attack may occur, an event characterized by nonstop tics of higher severity that often incapacitate the physical motion and verbal communication of the patient. The frequency of tic attacks can range from occasional (less than once a month) to daily; attacks can vary dramatically from person to person, and they can last from a few minutes to several hours [4]. There is no cure for tics or tic attacks, but the severity of tic attacks can potentially be reduced by medications such as neuroleptics or fluphenazine [5]. Providing medicine and emotional support is typically the job of caretakers, such as parents, guardians, and nurses, but constant surveillance is not always possible.

### Product/Need

Few products currently exist for patients with TS. There are some products designed to mitigate tics through slight electrical pulses, but these do not help solve the problem of detection and alerting someone for help. TSBand is a wearable wrist device that aims to automatically detect when a tic attack is occurring and then alert a nearby caretaker with a mobile notification. The device serves as a bridge between caretakers and patients, allowing those in need to receive help without physically needing to call for it. This can potentially decrease the span of a tic attack from hours to just a few minutes with help from the caretaker, limiting the time of struggle and the possibility of self-harm. This is helpful for adults but is especially helpful for children, who are more inexperienced with handling attacks, as caretakers can be notified instantly via TSBand and help the child by quickly practicing behavioral techniques to calm the child down, reducing the time of the attack. Additionally, studies have shown that medical technologies integrating mobile phones and wearable sensors need improvement and further scrutiny [6]. This paper proposes the development of TSBand to detect tic attacks and notify caretakers.

## Methods

### Sensors Used for Detection

To facilitate the detection mechanism of the device, common patterns that people with TS usually exhibit must be analyzed. TSBand is equipped with a triaxial gyroscope and accelerometer, a pulse sensor, and a body temperature and humidity sensor. When tic attacks occur, patients usually exhibit uncontrollable and repetitive upper limb movement. The gyroscope and accelerometer help in providing data to perform the necessary computations to determine tic attacks from arm movement. The accelerometer is used to gather data on the speed and acceleration of the arm during tic attacks, while the gyroscope is used to provide information on roll, pitch, and yaw to account for the variation in angle and thus calculate the angular speed and acceleration. Furthermore, a microphone is used to assist with the detection and account for vocal tics as well (described in the Audio Analysis section).

In one previously published paper, researchers described a detailed clinical study showing that high blood pressure, stress, and an increased heart rate during tic attacks are all signs and symptoms commonly reported by patients with TS [7]. By using these vital signs in combination with movement, the tic detection algorithm can be enhanced for a higher degree of accuracy. To measure these factors and symptoms, TSBand includes a pulse sensor that actively measures the heart rate of the user. As temperature and sweat can increase because of both movement and stress, the body temperature and humidity sensor monitors for increasing fluctuations in these 2 vital signs. Combined, these sensors are used to detect changes in the vital signs of patients with TS and serve as another method to monitor for tic attacks.

### Machine Learning

Because tics and tic attacks vary from person to person, the detection model cannot be generalized, rather, it needs to be custom-tailored to each individual. To monitor for and detect tic attacks, TSBand requires a 2-day calibration period in which the user’s movement patterns are stored to determine the tic patterns of the individual patient. The wristband will need to be worn both during the day and at night for calibration, as severe tics persist throughout the night and during sleep in some people. The device calibration is used primarily to detect the regular movement patterns of the user to determine a common baseline and allow the wristband to adapt to the unique baseline of the patient. There does not need to be any tic attack during the calibration period for the algorithm to work as intended; if there are any tic attacks, the algorithm will likely classify them as outliers based on the other data collected during this period. Using the local outlier factor (LOF) algorithm, TSBand separates tic attacks from normal, everyday movements. The variables that the LOF model analyzes are speed and acceleration; these were chosen because traditional models such as random forest and regression are less accurate at detecting tic attacks with these parameters. LOF models use a vast amount of data to form clusters and, when optimized, can be used to find outliers in the data set.

LOF uses unsupervised machine learning techniques that use the density distribution and standard deviation of data points to detect outliers in the data. Considering reachability distance and the density of the data values, TSBand uses a fine-tuned algorithm to limit the nearest neighbor’s parameter to ensure that clusters will not be formed near a tic attack, helping reduce the number of false positives:




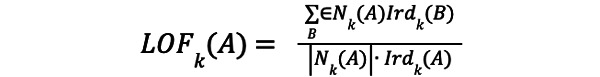




By obtaining data for 2 days to train the model, TSBand can uniquely tailor the detection system to each individual user for ease of customization and personalization. The 2-day calibration period also helps limit both the number of false positives that occur and the number of clusters around tic attacks, thus increasing the overall accuracy of the model. The data during the 2-day calibration are the only data used to determine outliers; all later data are not recorded or stored for detecting tic attacks.

In addition to the outlier algorithm, a linear regression model—used to find trends over data points—was implemented as a measure to enhance the detection process. The regression model measures data from the last hour to search for dramatic increases and changes in vital signs parsed through a certain threshold value. The threshold is passed when the rate of change at the data point is greater than or equal to 4/3 or less than or equal to –4/3. The ±4/3 threshold value was chosen because vital signs such as heart rate are usually stable, and when variations occur, the changes are dramatically visible, allowing ±4/3 to be a sensible rate of change to use. However, as this threshold range was chosen through a reasonable estimate, it is subject to change after refinement of the algorithms with patient testing:

y=b_0_+b_1_x_1_

Threshold Range: –43 ≤ y’ ≤43

The algorithm uses this principle to determine whether vital signs such as stress, sweat, body temperature, and heart rate are increasing or are predicted to increase for the wristband bearer. This mechanism can be used to find general trends in vital signs that are clear indicators of tic attacks. The combined algorithms output a percentage that conveys the likeliness of a tic attack occurring. Although variations in heart rate, temperature, and humidity might signify an attack, these parameters are not always correlated with a tic attack, while movements are. To place greater emphasis on movement, 70% of the final prediction relies on this parameter, while the other values equally share the remaining 30%. These ratios are subject to change after testing among real patients with TS is completed and data are aggregated:

P_Total_=0.7P_Movement_+0.1P_HeartRate_+0.1P_Temperature_+ 0.1P_Sweat/Humidity_

Once the probability of the total attack (P_Total_) is calculated, any result over 80% will register an alert and notify the caretaker. When the threshold is passed for the linear regression models, the attack is considered “true” (ie, P_Vital_=1), and the model will stop analyzing new data until the patient can confirm the attack is over on the mobile app or there has not been a tic attack registered (ie, P_Total_>80%) for an hour. When either of these conditions is met, the model will then assess the data from the last hour and continue monitoring new vital sign data from thenceforth. The value of 80% for P_Total_ is a default value and can be changed manually by the caretaker and the patient to suit their specific needs through our companion mobile app. This feature was added in consideration of people who may not experience large fluctuations in health vital signs during tic attacks; however, the value should remain above 70% so as to not rely only on movement.

### Band Design and Schematics

The wristband has a sleek design to enhance the experience of the user. The band is powered using an ESP32 Wi-Fi chip (Espressif Systems), which is used to send data to a live-updated database hosted on Firebase (Google Inc). To program the ESP32, the Arduino C++ language was used. All the sensor data collected from the user are securely stored in Google’s real-time Firebase server, where the algorithms, hosted on Google Cloud, execute decisions and determine whether a tic attack is occurring. The wristband may also potentially be able to predict tic attacks before they occur. Although movement cannot be used as the sole factor for prediction because stress is often a precursor to tic attacks, increased heart rate and body temperature can potentially be used as a predictive mechanism. When a tic attack is detected, an alert is immediately sent to the caretaker’s phone through the companion mobile app. A buzzer is built into the band that sounds to act as an audio cue to notify the user that an alert has been sent.

**Figure 1:**
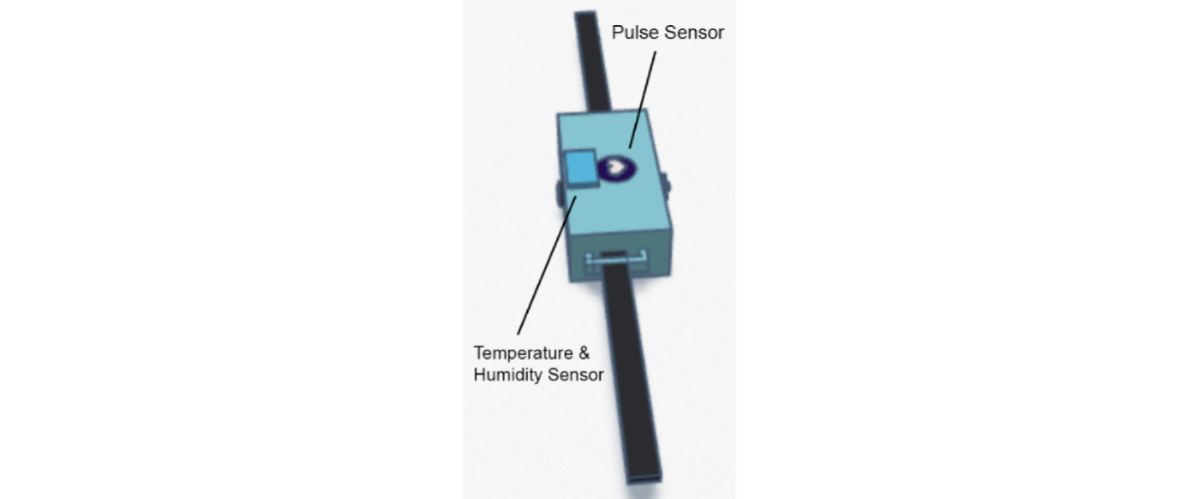
Computer-aided design model of the TSBand prototype (bottom view).

**Figure 2:**
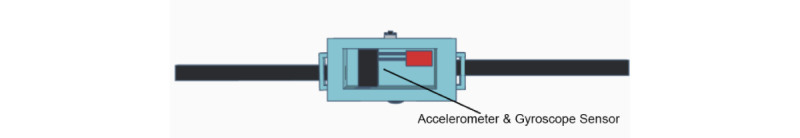
Computer-aided design model of the TSBand prototype (bottom view inside the compartment).

**Figure 3:**
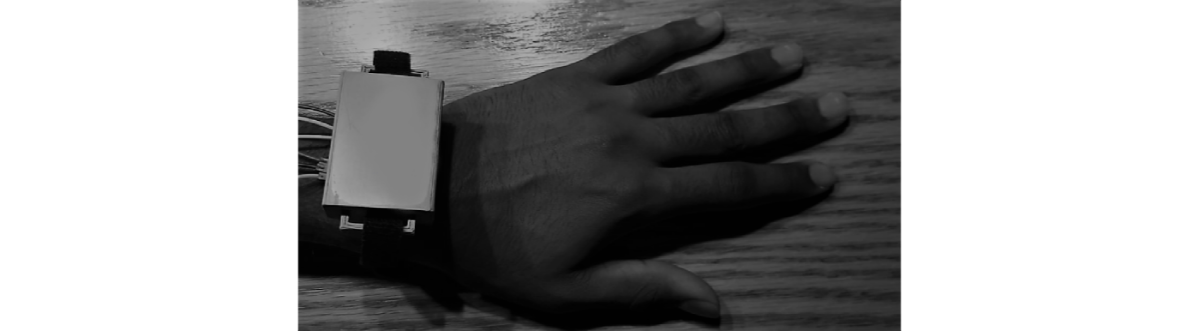
TSBand prototype being worn on the wrist.

A backup method exists in case a tic attack occurs but the algorithm does not detect the attack; this allows the user to manually send an alert to the caretaker. A button on the device can be pressed and held for 5 seconds to send a request to the caretaker for help. A potential issue with the button is that a user could develop a motor tic and press the button without the intention to call a caretaker for help. Therefore, the button must be held for 5 seconds to give the user time to reconsider the action and let go of the button if necessary. Additionally, a cancellation feature was implemented in case a faulty alert was sent for any reason. If the user notices the buzzer going off, they can press the button 3 times (or more) in the span of 1 second to cancel the request to the caretaker. Figures 1 and 2 show a computer-aided design model of the prototype, and Figure 3 displays the prototype being worn. Because of budget constraints, we were not able to scale down the size of our central processing unit, which is why wires can be seen coming out of the device in Figure 3.

### Audio Analysis

Tics do not only occur in the form of motor movements—they can also appear as verbal noises. Vocal tics are common and words or specific phrases are typically blurted out at random moments. During tic attacks, patients with TS usually repeat those specific phrases or other sounds over a very short period. On the device, audio files obtained through a microphone are passed through a high-pass filter to limit background noise. By converting speech to text and analyzing patterns in speech over a certain time interval, the wristband is able to detect and process vocal tics. The model used to train the speech recognition software is a recurrent neural network model that uses a long short-term memory algorithm. The data were compared with the Google Speech data set and had an accuracy rate of 92%. To limit the amount of error in this trial, the algorithm was designed to listen for and catch the frequency of a specific word repeated over a period of 10 seconds. If the repetition occurs for a duration that is longer than average for the user, a notification is sent to the caretaker. This audio detection mechanism is independent of the physical detection mechanism; if one detects an attack while the other does not, an alert will still be sent.

**Figure 4:**
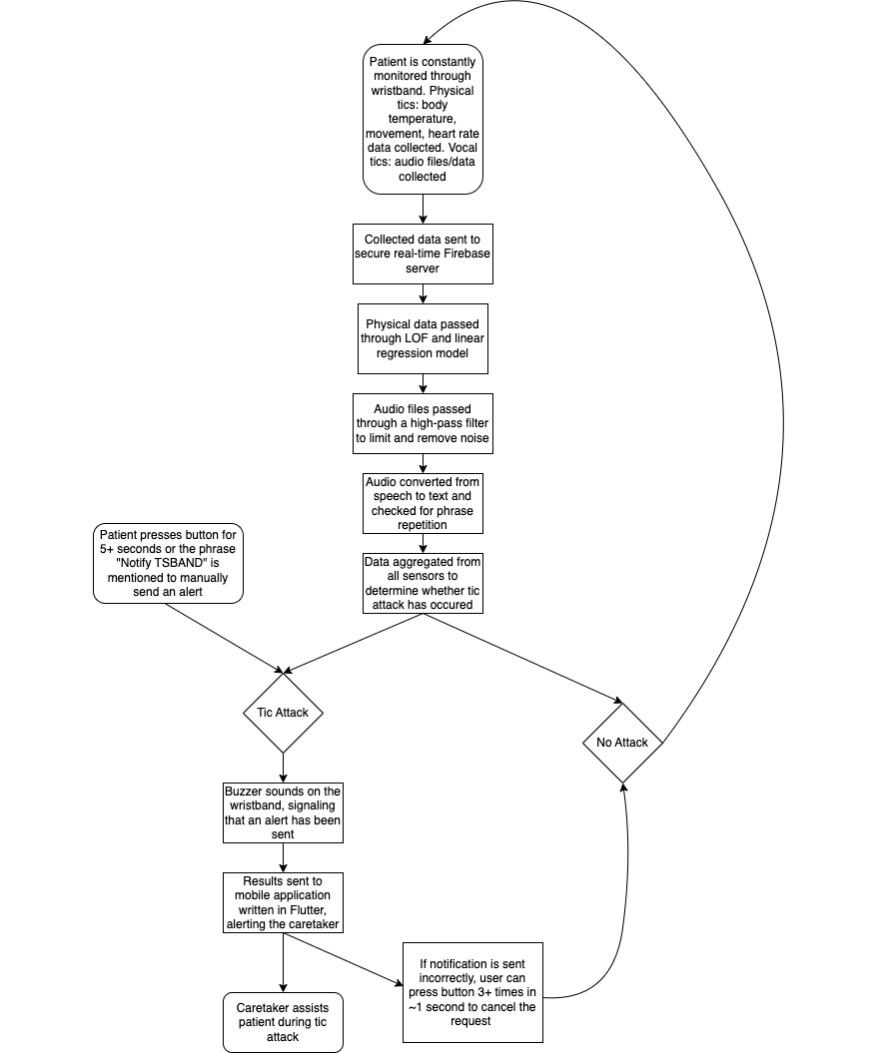
Flowchart diagram and schematics of the TSBand in operation. LOF: local outlier factor.

**Figure 5:**
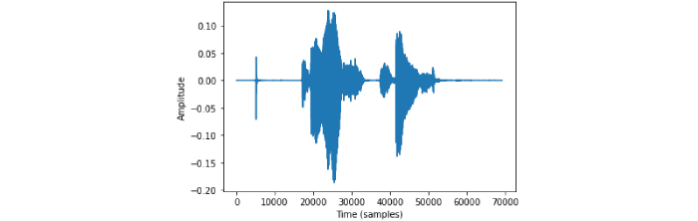
“Notify TSBand” vocalization signal diagram.

**Figure 6:**
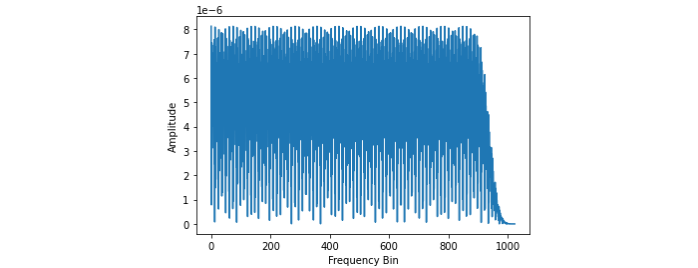
TSBand spectrum diagram.

**Figure 7:**
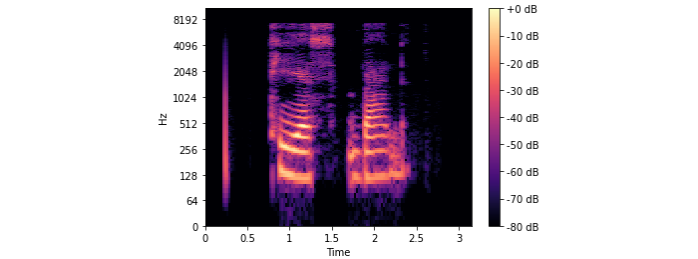
TSBand spectrogram diagram.

**Figure 8:**
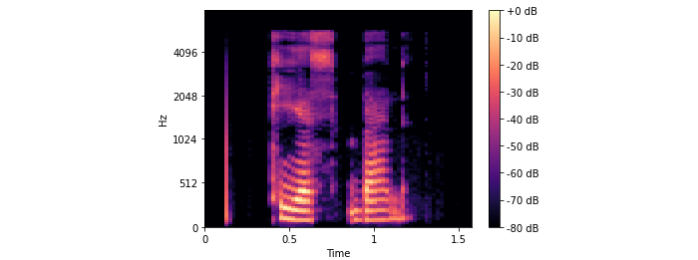
TSBand mel spectrogram diagram.

Furthermore, a backup vocal alert system is also included in the band as a supplement to the audio analysis. Much like smart home devices such as Amazon Alexa or Google Home Mini, a prerecorded audio cue phrase can be set to instantly send an alert notification to the caretaker. For example, if the cue phrase is “notify TSBand,” a notification is sent to the caretaker alerting them of an ongoing attack any time the wristband detects this specific phrase. The phrase only needs to be said once, which is why it is important to pick a specific, uncommon phrase. This is intended for an absolute emergency backup case, such as when the automatic detection and repeated phrase detection fail and the backup button is not pressable due to an excessive number of tics that limit movement. There is potential that the user develops a regular verbal tic for this exact phrase, causing the alert to be triggered when there is no tic attack. Thus, the audio phrase that triggers the alert can be changed and prerecorded again through the mobile app to preserve the use of this feature. Again, the buzzer will sound to notify the user that an alert has been sent, and the cancellation feature is still valid in this scenario if it is necessary to cancel the request. The flowchart in Figure 4 summarizes the process. Figure 5 shows the vocalization diagram of the phrase “notify TSBand” in a signal diagram. Figure 6 shows the vocalization in a spectrum diagram. Figures 7 and 8 show the audio vocalization under a spectrogram and a mel spectrogram, respectively.

### Mobile App

TSBand has a companion mobile app to send notifications to caretakers. After downloading the app, a caretaker or a group of caretakers can pair with the wristband—this can be done as each band has a unique hash code associated with it that can be used to accurately pair up with the app. The app displays certain health vitals, namely heart rate and body temperature, and an audio monitor that shows the frequency of words and reports if there is anything abnormal. The value is shown as either “stable” or “unstable” depending on the count of the repetition of words (it is determined the same way as described in the Audio Analysis section). Heart rate and body temperature are read directly from Firebase and are displayed live on the app.

**Figure 9:**
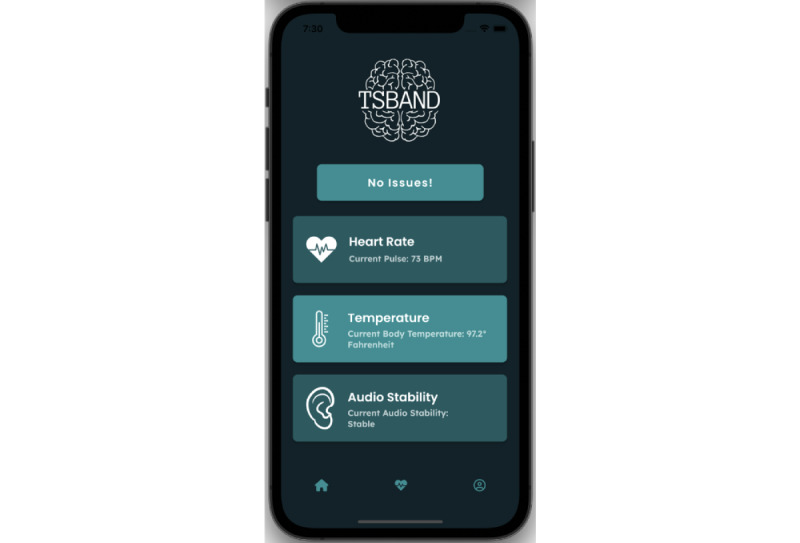
Mobile app when tic attack is not occurring.

**Figure 10:**
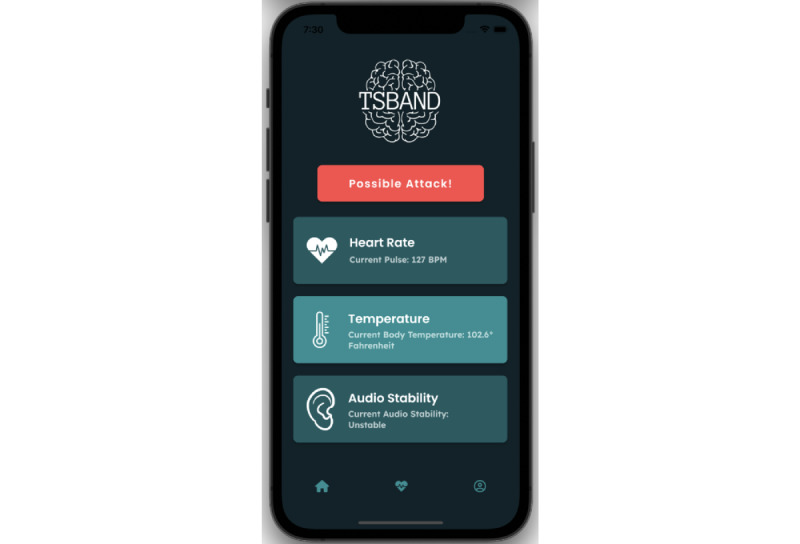
Mobile app when tic attack is occurring.

As shown in Figure 9, typical activity will result in the bar at the top showing the status “no issues.” If a tic attack is detected by the algorithm, a push notification (pop-up notification) will appear on the phone to alert the caretaker. Upon opening the app, as shown in Figure 10, the “no issues” bar at the bottom will have changed to the “possible attack” status. The app will not send any new push notifications on the phone while the status is “possible attack.” If the situation has been handled, the “possible attack” button can be pressed on the app to revert the status back to the normal “no issues” value, and at that point, the app will once again send push notifications in case another tic attack happens. Pressing this button will lead to the restart of the regression models.

### Additional Procedure

We have not yet had the opportunity to evaluate or test our device with physical participants or patients due to funding and time constraints. This paper focuses on introducing the technology behind the device and analyzes its potential introduction into the market. We have performed said analysis through a preliminary research survey to obtain perspective on the potential of the device. This survey—mainly consisting of questions inquiring about the personal experiences of tic attacks among patients with TS and asking for feedback and new features for the wristband—was filled out by 70 people with TS and is detailed in the sections below.

### Ethical Considerations

The survey did not contain any questions that would induce physiological stress or anxiety and collected only nonsensitive data; thus, we did not need ethics approval. A link to the quantitative questions and results is available in the Data Availability section.

## Results

### Finances

For the economic and financial analysis, we determined the current cost of our prototype in US dollars (Table 1), but since parts and materials are subject to change, we did not predict a potential final price. In the future, since the current prototype is bulky, we plan to further size it down. We hypothesize that the overall prototype cost will be reduced with mass manufacturing, but it is difficult to estimate the price of a smaller central processing unit and other final parts, as there are many variables involved with finding new components.

With a final cost of $62.74, TSBand will be suitable for potential users. Furthermore, on the mobile app side of this service, it is estimated that server upkeep and fees for application programming interfaces used for the software behind the device will cost an additional US $5.00 per user per year. If the wristband is mass manufactured with a custom-printed circuit board, both the cost and size of TSBand can be reduced.

**Table 1:** Prototype cost structure.

Components	Cost (US $)
ESP32 feather	9.95
Pulse sensor	10.99
Humidity and temperature sensor (DHT11)	5.00
Gyroscope and accelerometer sensor (GY-521)	6.00
Audio microphone (AOM-5035L-HD3-R; PUI Audio)	4.45
Buzzer (WT-1614T; Soberton Inc)	1.25
Battery source (130 maH)	19.99
Velcro	0.11
3D printing	5.00
Total cost	62.74

### Research Survey

The questionnaire was an online survey consisting of 11 questions, both qualitative and quantitative, directly addressing the TS community. The survey was a completely blinded review survey performed online on social media platforms that posed both quantitative and open-ended questions. To ensure anonymity and to abide by Health Insurance Portability and Accountability Act and the General Data Protection Regulation, data on the participants were not tracked so that responses would be more transparent. The collected data are held securely.

### Survey Findings

Notably, 64 of 70 (91%) respondents said that they struggle to communicate with caretakers when having a tic attack, and 67 of 70 (96%) respondents believed that stress and anxiety are contributing causes of tic attacks. In qualitative responses, one common comment was that an alert could help bring forth a trusted caretaker who could help calm the patient down and ensure they do not cause self-harm. Some mentioned that the material of the wristband is also extremely important due to skin irritation that some face. In addition, the structural integrity of the band must be strong to ensure longevity and so the band does not break when exposed to movement during an attack. We found that 30 of 70 (43%) respondents believed that having a person near them would help them cope with the attack, while 13 of 70 (19%) respondents said it would only help if the figure was trusted. Overall, 53 of 70 (76%) respondents stated that such a device would be useful for them. Those who stated that the device would not be beneficial to them were adults who did not have caretakers or did not use medication, as their tic attacks were of shorter duration.

## Discussion

### Limitations

Certain limitations still exist with wristband detection. For example, it is difficult to differentiate exercise from a tic attack, as both include excessive movement of the body. If the machine-learning model is not trained with exercise data, it may consider simple tasks like running to be a tic attack, but if the model is trained with exercises like running, it may potentially consider a tic attack to be another aspect of a daily routine and not trigger an alert. A model trained without exercise would force the user to take off the wristband during physical activity and leave them vulnerable to a sudden tic attack with no automatic detection. Ideally, someone else would supervise them in this scenario, but adding measures such as the ability to turn off the automatic detection algorithm of the band while still keeping backup elements active would be optimal for users when exercising. Alternatively, if the model is trained with exercise, there is potential that with vast data on previous patterns of the patient and an exercise mode built into the wristband, the algorithm can stay powered on during activities like running and assess differences between tic attacks and exercise. Creating an exercise mode would need much experimentation and previous data on the user’s personal tic attacks to accurately differentiate between physical activity and tic attacks. We also recognize that the survey of 70 respondents does not represent the entire population of people with TS.

### Comparisons to Similar Wristbands

Similar studies have been conducted for tic movements and other tremors, such as those caused by Parkinson disease (Table 2).

**Table 2:** Comparison with similar wearable technologies.

Paper	Project purpose	Measure of movement	Measure of vital signs	Recognition method	Audio analysis
This paper	Monitor for and detect tic attacks	Accelerometer, gyroscope	Heart rate, body temperature, sweat	Local outlier factor, regression, threshold	Long short-term memory
Bernabei et al [8]	Detect tic movements	Accelerometer	None	Time variant threshold	None
Fraiwan et al [9]	Monitor Parkinson tremors	Mobile phone accelerometer	None	Deep-learning neural networks	None
Kim et al [10]	Differentiate upper limb tremors from regular movement	Accelerometer, gyroscope	None	Statistical pattern recognition	None
Cole et al [11]	Detect tremor and dyskinesia in Parkinson patients	Accelerometer	Surface electromyography	Support vector machine and Markov models	None

The most similar wristband to TSBand is the band developed by Bernabei et al [8], which uses a triaxial accelerometer to determine tic movements. It corroborates video analysis with the wristband data to detect tic activities, but it cannot detect tic attacks. Furthermore, it does not use any other factors, such as angular acceleration, vital signs fluctuations, or audio analysis to determine tic movements, and is not tailored to each individual that uses it. Kim et al [10] applied an inertial measurement unit on a worn device to differentiate typical daily movement from tremors in the upper limbs. Artificial tremors were generated, and the data were passed through statistical pattern recognition to determine if a reading was a tremor or standard activity. The studies conducted by Fraiwan et al [9] and Cole et al [11] both monitored for tremors in Parkinson patients, with the former using the accelerometer in a smartphone and the latter using a separate accelerometer sensor. Both used neural networks for the detection of tremors, but Cole et al also used surface electromyography for a higher rate of accuracy. The machine-learning techniques used in both studies were used only for tremor detection accuracy and did not incorporate customizability for individual patients, as all Parkinson tremors are quite similar. The machine learning models used in TSBand, however, are intended to adapt to each person’s unique tics and tic attacks for better detection purposes. This wristband serves as a complete solution that could immediately be used by those in need, with backup and emergency situations accounted for in its design. Because of the personalization TSBand provides, it could also potentially be used for similar conditions with similar symptoms, including myoclonus, tremors, chorea, athetosis, dystonia, akathisia movements, paroxysmal dyskinesias, and ballistic movements.

### Future Work

Initially, TSBand was designed to be a thin bracelet strapped around the wrist, but because the sensors took up a large amount of space, a storage compartment was added to hold them. However, this storage compartment now gives the potential to add a light emitting diode screen, among other options, on top of the compartment, which could be used to enhance the user experience. Based on survey responses, certain features could be added to the band and mobile app to improve user benefit. One common suggestion was a GPS system to determine the location of the attack to better notify the caretakers, as well as a screen on the wristband itself that shows how far away the caretaker is to give an estimate of how long it would take for them to arrive. Another feature was for the app to not only notify caretakers, but also inform teachers or coworkers that a tic attack is occurring. Several individuals who were students mentioned that they were embarrassed to raise their hands to tell teachers that they needed to leave the classroom during a tic attack, so this mechanism would effectively avoid said embarrassment by providing a tacit signal to the teacher that the user needs some time and space away from class to calm down.

We would like to test the current accuracy of the band among patients with TS to determine specific values for the regression model, see which areas need to be improved on, and understand how new features can be incorporated (eg, the exercise mode). A common request from the survey was a way to also send information on the severity of the attack so as not to worry the caretaker if the attack is only mild. Adding this would be extremely helpful but would require a great deal of testing and experimentation to measure severity. We are also looking to enhance our algorithm to potentially predict tic attacks before they occur based on previous behavior patterns and also scale down the size and cost of TSBand through a custom-printed circuit board to fit better on the wrist. In terms of sensors, upgrading the band to include more accurate and precise sensors, such as galvanic skin response and blood pressure sensors, would allow it to detect tic attacks more effectively and accurately. We filed a provisional patent with the United States Patent and Trademark Office on October 8, 2022, and we hope to finalize these upgrades and finish conducting this research by September 2023.

### Conclusion

In this work, machine learning and audio analysis are used to detect tic attacks. LOF is used to individualize the detection process, with a regression model used to ensure greater accuracy. To reiterate, we were not able to test these machine learning components with actual patients due to funding and other concerns; we aim to do this in the future. Audio analysis is used to check for repeated phrases over a time interval to detect vocal tics. In addition to these features, we also have an emergency audio backup method along with our standard backup button. A recurrent neural network was trained on a Google Speech data set to obtain a 92% speech accuracy rate. Within the next year, we hope to add new features, scale down the size of the device, add new sensors, and test the wristband among patients with TS. We conducted a survey involving 70 participants with TS to gather data on commonalities in tic attacks, the efficacy of having a caretaker nearby, and qualitative feedback to help determine limitations and solicit suggestions on the development of the wristband. The benefit of a platform that connects patients at risk with caretakers is not limited to those with TS. The elderly or people at risk of seizures can benefit from having an alert automatically sent to a caretaker when they are not able to physically move or request help themselves. Any person that requires an easy method of requesting assistance can use this wristband and receive help faster. This wristband has vast potential and can not only be applied to TS but used in many different fields of health care and for patient treatment.

## Abbreviations

GDPR: General Data Protection Regulation
HIPAA: Health Insurance Portability and Accountability Act
LOF: local outlier factor
TS: Tourette syndrome

## Data Availability

The data sets generated during and/or analyzed during this study are available in the Open Science Framework repository [12].
